# From authorisation to clinical practice: evolution of the use of biological medicines according to the SmPC and guidelines (2006 to 2025)

**DOI:** 10.1007/s00228-026-04097-5

**Published:** 2026-06-10

**Authors:** B. M. F. Penninx, C. E. M. Hollak, S. W. Tas, L. Timmers, S. J. de Visser, Z. L. E. van Kempen

**Affiliations:** 1 Medicine for Society, Platform at Amsterdam University Medical Center, University of Amsterdam, Amsterdam, The Netherlands; 2 Department of Endocrinology and Metabolism. Amsterdam University Medical Center, Amsterdam Gastroenterology Endocrinology Metabolism (AGEM) Research Institute, Expertise center for inborn errors of Metabolism, MetabERN, University of Amsterdam, Amsterdam, The Netherlands; 3 Department of Rheumatology & Clinical Immunology, Amsterdam Rheumatology & Immunology Center, Amsterdam University Medical Center, University of Amsterdam, Amsterdam, Netherlands; 4 National Health Care Institute (Zorginstituut Nederland), Diemen, The Netherlands; 5 Erasmus School of Health Policy and Management, Rotterdam, Netherlands; 6 Centre for Future Affordable and Sustainable Therapy development (FAST), Leiden, the Netherlands; 7 Department of Neurology, Amsterdam University Medical Center, Vrije Universiteit, Amsterdam, The Netherlands

**Keywords:** Biological, Efficacy-effectiveness gap, Clinical guidelines, SmPC, Post-marketing studies

## Abstract

**Purpose:**

After the marketing authorisation of biologicals, uncertainty often remains regarding their real-world effectiveness, safety, and rational use. This study investigates how frequently the recommended use of biologicals changes post-authorisation, and who funds the studies leading to these changes.

**Methods:**

Biologicals receiving a positive opinion for adult indications by the Committee for Medicinal Products for Human use (CHMP) between 2006 and 2010 were identified. For each indication, we compared the initial and current Summary of Product Characteristics (SmPC), as well as current SmPC and European clinical guidelines, to identify discrepancies in start and stop criteria, dosage, and administration frequency. We assessed whether discrepancies led to increased or decreased use of the biological and whether this was associated with the sponsor type of supporting publications.

**Findings:**

SmPC changes, driven by Marketing Authorisation Holder (MAH)-sponsored studies, were found in 47% of identified indication-biological combinations: 41% led to increased use, 29% to decreased use, and 29% were neutral. Guideline-SmPC discrepancies, mostly supported by publicly sponsored studies, were identified in 75% of cases, with most discrepancies (86%) recommending decreased use. Publications sponsored by public healthcare stakeholders were associated with decreased use, as compared to publications sponsored by the MAH (*p* < 0.05, OR 28·2, 95% CI 4·8–166·1).

**Conclusion:**

Recommendations for the use of biologicals often evolve post-authorisation. MAH-sponsored studies tend to support increased use, whereas guideline recommendations, typically based on publicly sponsored studies, more often favor restriction. Rational use of biologicals requires timely evidence generation and should ideally lead to amendments in SmPC’s.

**Supplementary Information:**

The online version contains supplementary material available at 10.1007/s00228-026-04097-5.

## Introduction

Biologicals are drugs that are produced using living cells or organisms [1]. The authorisation of recombinant insulin in 1982, the first biological produced using recombinant DNA technology, marked the beginning of a new era in medicine by demonstrating for the first time that human proteins could be produced in bacteria [2]. Since then, advances in biotechnology have enabled the development of increasingly large and complex molecules capable of targeting proteins and cell surfaces that were previously inaccessible to small molecules, greatly expanding therapeutic possibilities. Consequently, biologicals have reshaped treatment and significantly improved patient outcomes in a variety of diseases, particularly those of inflammatory and oncological origin.

When medicines receive marketing authorization by the European commission, they are assigned a Summary of Product Characteristics (SmPC) that is based on the evidence available at the time of authorisation, which may evolve over time. A trade-off exists between late marketing authorisation with extensive knowledge on clinically relevant outcomes and early authorisation with limited evidence. Since the 1990s, the era that marked the rise of biologicals, regulators have increasingly granted authorisations based on surrogate endpoints, small sample sizes, and single-arm studies [3–5]. While this may have led to earlier access to new therapies, it has also increased the gap between the recommended use at marketing authorisation and the safest use in practice [6, 7]. In addition, less robust evidence at authorisation has heightened uncertainty and widened the efficacy-effectiveness gap [8, 9]. Following marketing authorisation, emerging evidence gradually reduces uncertainty about real world effectiveness and informs safer and more rational use of biologicals. This may involve dose adjustments, changes to the frequency of administration, or modifications to the start and stop criteria of the biological. Even for biologicals with proven efficacy in well-conducted large randomized clinical trials, optimization opportunities frequently remain, for example because dose-finding studies often target the maximum tolerated dose to avoid missing potential treatment effects in trials [10]. Simultaneously the innovative nature of biologicals frequently translates into significant costs [11]. As a result, high prices are paid for medicines whose most rational use is still to be defined.

Rational use of medicines according to the World Health Organization (WHO) is when “patients receive medications appropriate to their clinical needs, in doses that meet their own individual requirements, for an adequate period of time, and at the lowest cost to them and their community” [12]. Although the scientific community has placed an increased emphasis on clarifying the most rational use of medicines after marketing authorisation [13], little is known about the evolution of the recommended use of biologicals during their real world use. A study examining biologicals approved between 2007 and 2014 by the European Medicines Agency (EMA) found that the dose or dosing frequency was altered after marketing in 11% of cases. The authors provided examples of dose reductions included in clinical guidelines that were not reflected in the SmPC, suggesting that the reported figure likely underestimates the extent of dose alterations in clinical practice [14]. A possible explanation for this discrepancy may be that only the Marketing Authorization Holder (MAH) can request SmPC changes and is not required to incorporate post-marketing evidence unless safety concerns arise. Moreover, in clinical practice, treatment guidelines play a dominant role in prescription behavior compared to the SmPC. We hypothesize that independent scientific evidence does not consistently translate into updates in the SmPC, while such evidence is incorporated more often into clinical guidelines.

Therefore, the objective of the current study was to evaluate how frequently SmPC changes occurred for indications for biologicals receiving positive opinions for marketing authorisation by the EMA between 2006 and 2010. The time frame was chosen to ensure observation of both early and delayed changes in recommended use. Furthermore, we investigated how the recommended use for these indications differed between current European guidelines and the most recent SmPC up to 2025. We examined four domains of recommended use, which were dose, frequency, start- and stop criteria. For each identified change in recommended use, the sponsors of the supporting evidence were explored to assess which stakeholders were primarily engaged in optimizing the use of biologicals.

## Methods

### Indication-biological combinations

Biological medicinal products that received a positive opinion of the Committee for Medicinal Products for Human Use (CHMP) of the EMA between 2006 and 2010 were identified from the CHMP meeting highlights. We included both newly authorised products and earlier authorised products receiving an additional approved indication in this period. The analysis concerned the indication-biological combination: a single biological could be included multiple times if it received positive opinions for multiple indications during the study period. Biologicals were defined as original, systemic therapeutic proteins or peptides that were produced with biotechnological methods, that is, derived from living organisms or their products. Biosimilars, vaccines, diagnostic proteins, cell-therapies, gene therapies, mRNA therapies, local therapies, and small molecules were excluded. Furthermore, indication-biological combinations were excluded from the analysis if the indication lacked an applicable European guideline. Biologicals with a withdrawn marketing authorisation per March 2025 were also excluded.

### SmPC changes and fuideline-SmPC discrepancies

For each indication-biological combination included, the four domains of recommended use (dose, frequency, start and stop) in the SmPC at introduction, the current SmPC and the current European guideline were obtained. The SmPC was obtained from the Union Register of medicinal products for human use. The SmPC at introduction was the first SmPC following notification of positive opinion by the CHMP. The current SmPC was last searched in March 2025.

European guidelines published in English were identified through the websites of relevant European societies. If no guideline was available, we searched PubMed for guidelines, consensus papers or statements published or endorsed by European professional societies or consortia. The search strategy is provided in the supplementary data. We included the most recent version of each guideline, last searched in March 2025. If a guideline did not mention a specific domain of recommended use, this was considered as no discrepancy.

Differences in the four domains of recommend use were identified between the SmPC at introduction and the present SmPC (hereafter: SmPC changes) and the present SmPC and the current guidelines (hereafter: Guideline-SmPC discrepancies). The differences were categorized by the direction of change (increased or reduced use of the biological for the specific indication). Only when the text of the included indication-biological combination in Sect. 4.1 of the SmPC, changed over time, was this interpreted as a change to the recommended start criteria. Section 4.1 of the SmPC includes the therapeutic indications for which the marketing authorisation has been granted by the European Commission. The approval of a new indication in Sect. 4.1 was treated as a separate indication–biological combination and not as increased use. Recommended dose, dosing frequency and stop criteria (when available) were identified from Sect. 4.2 (posology) of the SmPC. Changes in the stop criteria were not included if they referred to unacceptable toxicity, disease progression (for (hemato)-oncological indications), or lack of response without clearly defined criteria. These stop criteria are presumed to be already implemented in clinical practice with or without specific mentioning in the SmPC.

### Supporting evidence

Evidence supporting recommendations underlying SmPC changes was identified from Sect. 5.1 and 5.2 (pharmacodynamic and pharmacokinetic properties) of the SmPC, whereas evidence supporting SmPC–guideline discrepancies was identified from the relevant clinical guidelines. All cited studies, regardless of study type, were included when their findings were extrapolated to support a recommendation that was applicable to that specific indication and biological, even if the study did not directly investigate the indication-biological combination. This could for example occur when a study was conducted in a biological of the same class for the same disease and the guideline made no distinction between biologicals in formulating the recommendation. For cited systematic or scoping reviews and guidelines from other professional societies, we searched the manuscripts for references that supported the recommendation. If a systematic review included a meta-analysis or case series aimed at assessing the incidence of adverse events, we relied on the meta-analysis or case series itself rather than its individual references, as the aggregation of evidence was considered essential for the recommendation. The funding sources of the included publications were identified and categorized as the MAH, public healthcare stakeholders or co-funding between the MAH and public healthcare stakeholders. These stakeholders include academia, charities, patient organizations, government funding bodies, and health insurers. Publication years were extracted from the articles.

Statistical analyses were performed using R version 4.4.2 (R Core Team, 2024). Descriptive statistics were used to summarize the time between authorisation by the European Commission (EC) and publication of the supporting study. Normality was assessed using histograms and the Shapiro-Wilk test. For non-normally distributed data, the median and interquartile range (IQR) were reported; for normally distributed data, the mean and standard deviation (SD) were reported. To assess whether indication type (cancer vs. non-cancer) was associated with more SmPC changes and Guideline-SmPC discrepancies, Mann–Whitney U test analyses were performed for each outcome and their sum. Associations between the funding source of the publication ((co)-funding of the MAH vs. funding by public healthcare stakeholders only) and direction of change in recommended use (increase vs. decrease) were assessed using the Chi squares test.

## Results

### Indications

A total of 71 indications for biologicals in adults that met our definition received a positive opinion by the CHMP between January 2006 and December 2010. Of these 71 indications, a total of 53 were included. Excluded indication-biological combinations and their reasons for exclusion, lack of a European guideline (*n* = 7) or market authorization withdrawal (*n* = 11) are presented in supplementary Table 1. Of the included indication–biologic combinations, five (9%) were authorised as orphan medicines and one (2%) received a conditional marketing authorisation. The included indication-biological combinations and the guideline that was used to assess the recommended use are available in supplementary Table 2. Of the 53 indication-biological combinations, 17 (32%) were cancer indications; the remaining 36 comprised various non-cancer indications.

### SmPC changes

**Fig. 1:**
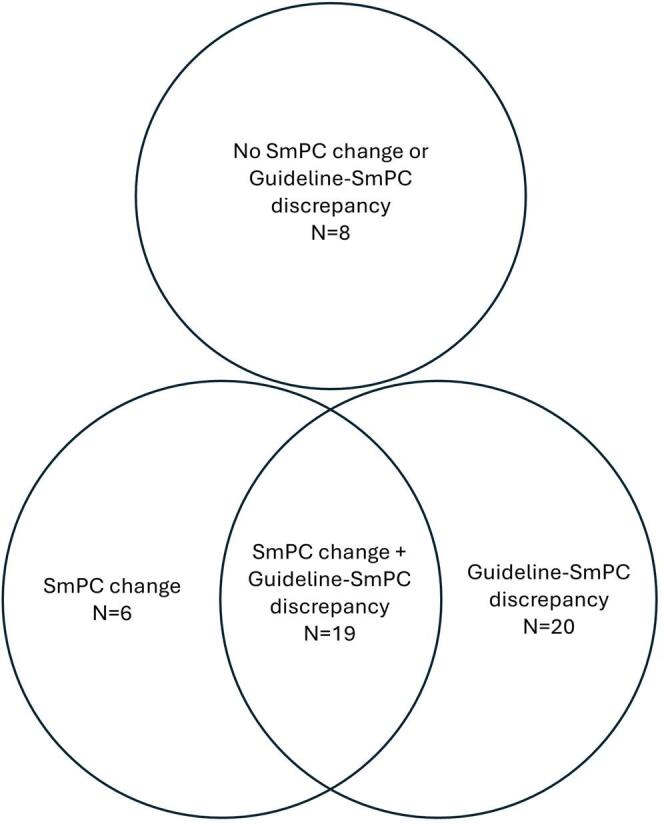
SmPC changes and Guideline-SmPC discrepancies of included indication-biological combinations

**Fig. 2:**
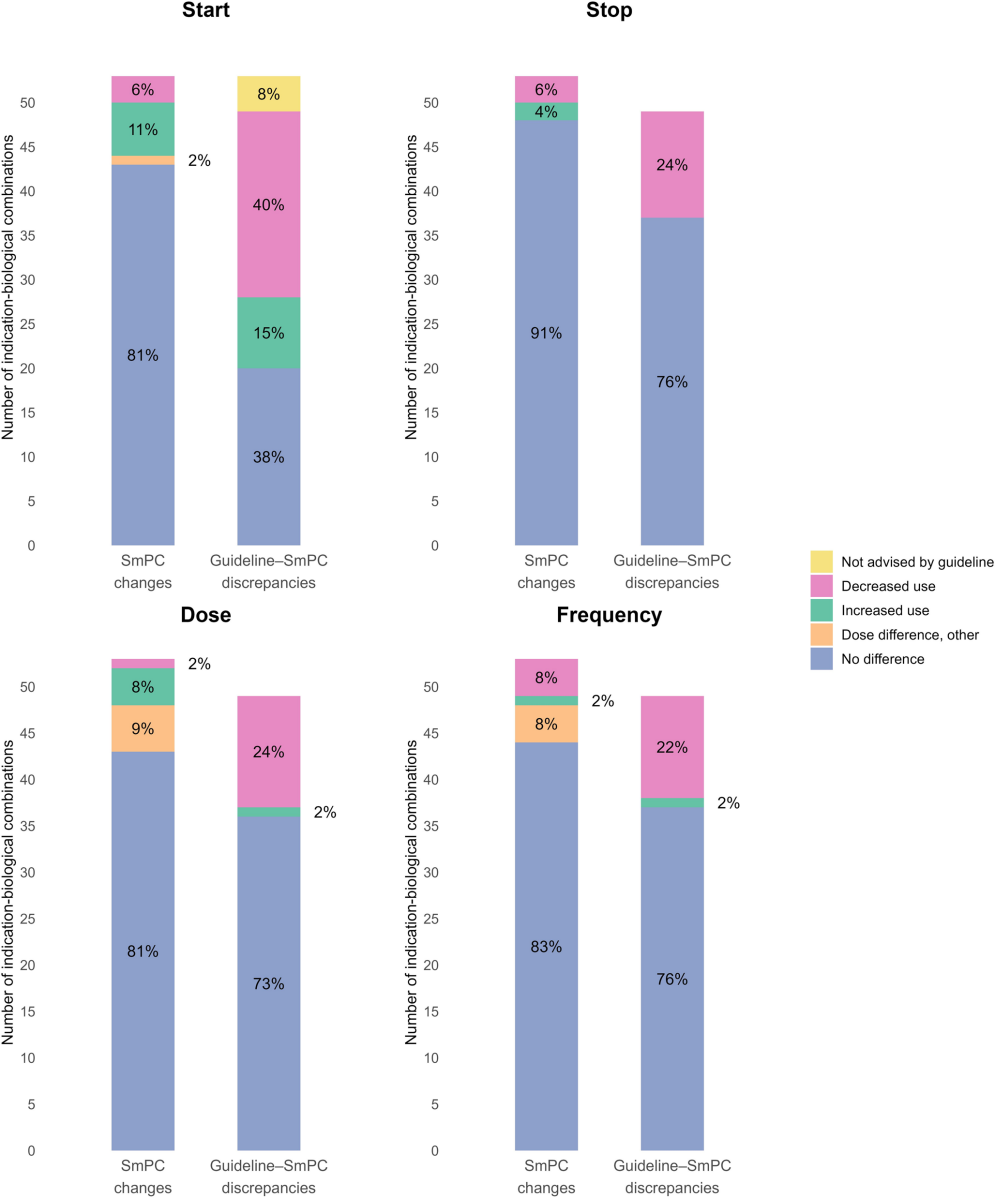
SmPC changes and Guideline-SmPC discrepancies and their direction. For some changes and discrepancies, it was not possible to determine whether they led to increased or decreased use of the biological. These changes or discrepancies are categorized as “Difference, other.” Only guidelines that advised the use of the biological for the specific indication were assessed for the domains dose, frequency, start- and stop criterium

We compared the SmPC at the time of introduction with the current SmPC for 53 indications. In total, 34 SmPC changes were identified in 25 (47%) indication-biological combinations (Fig. 1). Eight indication-biological combinations underwent two or more SmPC changes. Ten (29%) SmPC changes led to decreased use of the biological, 14 (41%) led to increased use of the biological and for ten (29%) changes no clear direction could be determined due to differences in dosing schemes with the same cumulative dose or due to individualization of dosing (Fig. 2). SmPC changes were less frequent in cancer than in non-cancer indications, although this was not statistically significant (median 0 [IQR 0] vs. 1 [IQR 1]; *p* = 0.07).

Of the 34 changes, five involved adjustments affecting multiple aspects simultaneously, such as changes to both dose and frequency or a stepwise reduction in administration frequency until discontinuation, and one involved adjustment of the stop criterion of a single biological for two indications. Each of these was treated as a single combined change for evidence assessment. This left 28 SmPC recommendations that were evaluated for supporting evidence.

### Guideline-SmPC discrepancies

In 39 of 53 (74%) European guidelines, 70 Guideline-SmPC discrepancies were identified. Sixteen indication-biological combinations showed more than two Guideline-SmPC discrepancies. Sixty (86%) Guideline-SmPC discrepancies led to decreased use of the biological, while 10 (14%) led to increased use of the biological (Fig. 2). Some guidelines included recommendations for multiple indication–biological combinations, for example recommendations about TNF-α inhibitors for psoriatic arthritis or bDMARDs for rheumatoid arthritis, and some recommendations affected multiple domains of recommended use. This resulted in a total of 37 guideline recommendations that were evaluated for supporting evidence. In Box 1, examples of SmPC changes and Guideline-SmPC discrepancies are described. SmPC–guideline discrepancies were less frequent in cancer than in non-cancer indications, although this difference was not statistically significant (median 1 [IQR 0] vs. 1 [IQR 3]; *p* = 0.07). In contrast, the combined total of SmPC changes and discrepancies was significantly lower in cancer than in non-cancer indications (median 1 [IQR 0] vs. 3 [IQR 2]; *p* = 0.01).

Box 1: Examples of SmPC changes and Guideline-SmPC discrepancies

**Table:** 

*Start (SmPC change)*: The start criterium in the SmPC of Methoxy polyethylene glycol-epoetin beta (Mircera) for Chronic Kidney Disease (CKD)-related anemia was changed from anemia associated with CKD to symptomatic anemia associated with CKD. *Stop (Guideline-SmPC discrepancy)*: For the prevention of hepatitis B virus (HBV) re-infection after liver transplantation, the SmPC of human hepatitis B immunoglobulin (Zutectra) recommends continued use of immunoglobulins in combination with an adequate virostatic agent. In contrast, clinical guidelines allow patients at low risk of HBV recurrence to receive only a short course of immunoglobulins or an immunoglobulin-free regimen, with long-term post-transplant prophylaxis continued as nucleoside or nucleotide analogue monotherapy. *Dose (SmPC change)*: In patients with Cryopyrin Associated Periodic Syndromes (CAPS), treatment with canakinumab is initiated stepwise until an adequate response is reached. The maximum dose in the SmPC was increased from 300 mg to 600 mg every 8 weeks. *Dosing frequency (Guideline-SmPC discrepancy)*: For the treatment of rheumatoid arthritis, the SmPC of rituximab describes repeated administration according to a fixed schedule. The European guideline in contrast, recommends tapering treatment in patients who achieve sustained clinical remission by gradually lengthening the dosing intervals.	

### Supporting evidence

Of the 28 SmPC recommendations that were evaluated for supporting evidence, in 23 occasions (82%) evidence was mentioned. Four SmPC recommendations were based on pharmacokinetic analyses, one on post-marketing surveillance data, and one on preclinical data; however, none of these sources were publicly available. For the 17 recommendations that cited traceable evidence, 19 publications in peer-reviewed journals were identified.

Of the 37 guideline recommendations evaluated for supporting evidence, nine (24%) mentioned such evidence. Fifteen (40%) substituted the biologic in the treatment algorithm with other treatments for (a part of) the specific indication without citing comparative evidence, 11 (29%) made recommendations without citing relevant evidence, and two (6%) explicitly stated that evidence for use of the biological within a specific subgroup consistent with the SmPC was lacking. For the nine recommendations that cited traceable evidence, 47 publications in peer-reviewed journals were identified.

**Table 1:** Sponsor of studies that led to increased or decreased use of the biological in the label or guideline

	Increased use (*N* = 17)	Decreased use (*N* = 47)
(Co-)Funded by the MAH (*N* = 30)	16	17
Public healthcare stakeholders (*N* = 31)	1	30

Studies that were co-funded by public healthcare stakeholders in collaboration with the MAH were counted as co-funded by the MAH. Studies that resulted in a negative recommendation for the biological in the guideline for that indication were classified as leading to decreased use

**Fig. 3:**
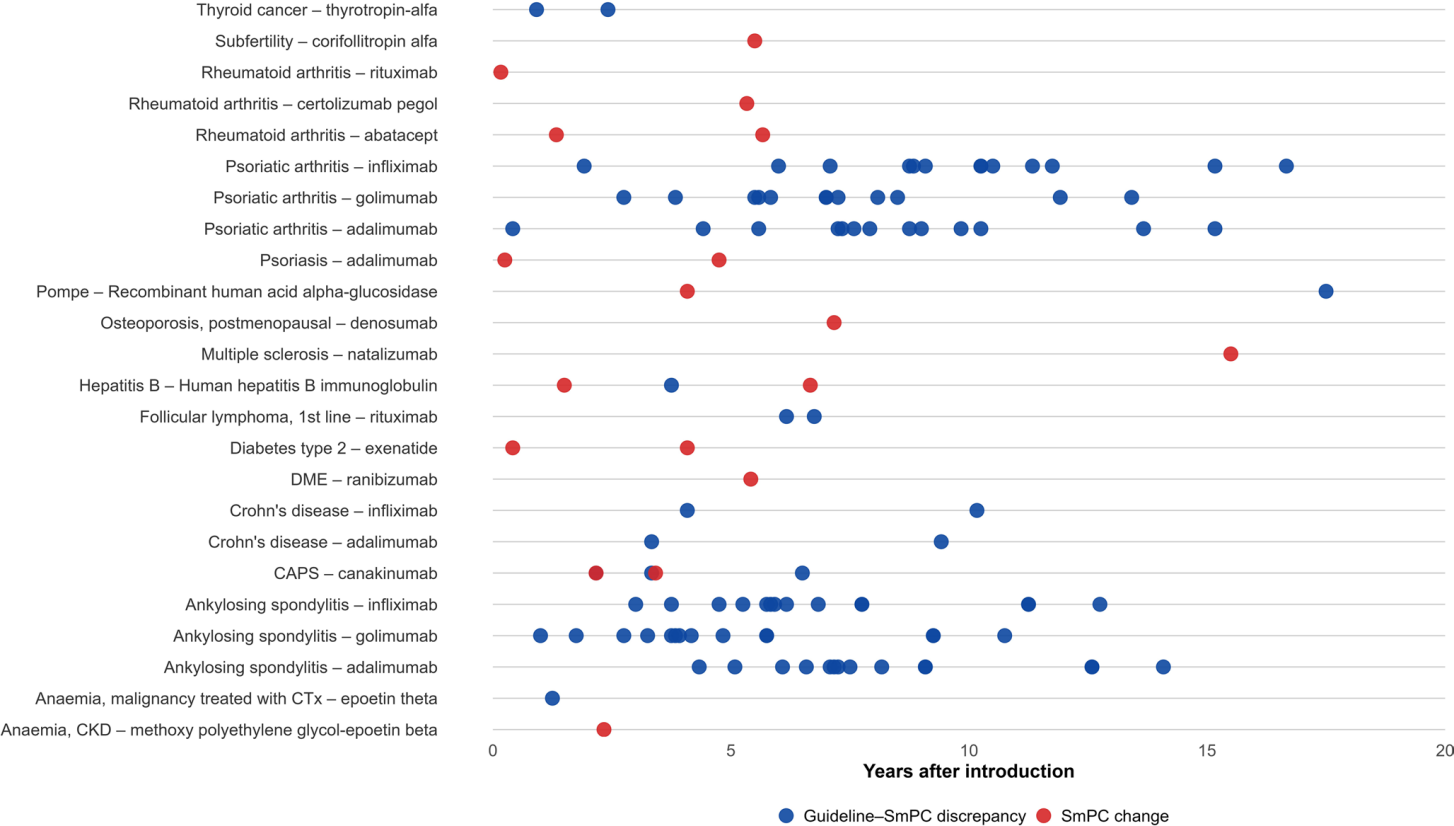
Identified publications that led to SmPC changes or Guideline-SmPC discrepancies and corresponding time passed since introduction. Indication-biological combinations without identified publications are not shown. A single publication may appear as multiple dots if guidelines made class-level rather than drug-specific recommendations. DME = Diabetic Macular Edema, CAPS = Cryopyrin Associated Periodic Syndromes, CTx = chemotherapy, CKD = Chronic Kidney Disease

Publications supporting SmPC changes were published a median of 49 months after marketing authorisation (IQR 20–65), whereas studies supporting Guideline-SmPC discrepancies were published after a mean of 87 months (SD 44). It is important to note that guideline recommendations were compared to the current label, which may partly explain the longer time to publication of supporting publications. Eight publications contributing to Guideline SmPC discrepancies and one supporting an SmPC change were published before marketing authorisation, and were therefore excluded from the analysis of median time from authorisation to publication and from Fig. 3.

All 19 publications that supported SmPC changes were funded by the MAH, one publication was co-funded with a public healthcare stakeholder. Of the 47 publications that supported a Guideline-SmPC discrepancy, 31 were funded solely by public healthcare stakeholders, 12 were funded by the MAH alone and four were co-funded by the MAH and public healthcare stakeholders. Table 1 presents the publications that contributed to either increased or decreased use of the biological, categorized by sponsor. A significant association was observed between the type of sponsor and the direction of use change (Chi-square test, *p* < 0.05). Compared to publications (co)-funded by the MAH, publications sponsored by public healthcare stakeholders were associated with decreased use (OR 28·2, 95% CI 4·8–166·1).

## Discussion

We evaluated SmPC changes over time and discrepancies between current European guidelines and the most recent SmPC of biological indications receiving a positive opinion by the CHMP of the EMA between 2006 and 2010 and followed up until March 2025. After a post-marketing authorisation period of at least 14 years, SmPC changes related to start and stop criteria, dose, and administration frequency occurred in 45% of the indication-biological combinations. When comparing the current SmPC to the current guidelines, 75% showed discrepancies. Figure 2 shows that these discrepancies occurred in all four domains (start, stop, dose, and frequency). Most guideline discrepancies led to decreased use of the biological for the specific indication as compared to the SmPC. These Guideline-SmPC discrepancies illustrate a gap between the use recommended in the SmPC and the current clinical use. As shown in Fig. 3, it may take a substantial amount of time after marketing authorisation before evidence informing SmPC changes or Guideline-SmPC discrepancies is published. Publicly funded studies were more likely to lead to reduced use of the biological. In contrast, sponsorship by the MAH was more often aligned with increased use of the biological, when indication expansion was not taken into account. This finding is consistent with previous research showing that non-mandatory, industry-sponsored post-marketing studies conducted within the approved indication tended to include broader patient populations than those defined in the original marketing authorisation [15]. 

This study also shows that the current SmPC does not always reflect the latest available evidence. As shown in Fig. 3, a substantial number of publications have informed the guidelines but have not been incorporated into the current SmPC. Article 23 of Directive 2001/83/EC of the European Union requires the MAH to provide the national competent authority with any information, including data from off-label use studies, that could affect the risk-benefit assessment of the medicinal product. Additionally, it mandates that the MAH must continuously update the product information to reflect the latest scientific knowledge [16]. Since the SmPC is perceived as a reliable and up-to-date source of information for prescribers, it is essential that its content is regularly updated. In this study, SmPC changes were exclusively based on studies sponsored by the MAH, with no SmPC changes linked to studies without funding by the MAH. To ensure the credibility of the label, mechanisms must be created that allow other stakeholders than the MAH to initiate SmPC updates based on independent research. The large number of Guideline-SmPC discrepancies and the tendency of MAH-sponsored studies to lead to increased use of biologicals may reflect fundamentally different objectives between public healthcare stakeholders and MAHs. Public healthcare stakeholders have little incentive to increase the use of medicines and may aim to contain costs while ensuring optimal care, and are therefore incentivized to conduct studies that promote rational use and to incorporate such evidence into guidelines. In contrast, MAHs have a commercial incentive to fund studies that expand use and thereby increase sales, and have limited incentive to update SmPCs based on publicly funded studies that lead to reduced use.

SmPC changes and SmPC–guideline discrepancies were less common in cancer indications. We hypothesize that this might reflect a lower tendency to study deviations from standard use in oncology, possibly due to concerns about irreversible consequences of undertreatment compared with non-oncological conditions. Importantly, the chosen time period predates the emergence of immunotherapies, which have since become a major therapeutic class in oncology. In recent years, there has been increased attention on improving the rational use of oncological therapies, driven by a growing focus on both toxicity and costs. For example, hybrid dosing of immunotherapy has been implemented on a large scale in the Netherlands [17], and the SONIA trial optimized the positioning of CDK4/6 inhibitors in the treatment sequence of hormone receptor (HR)-positive, HER2-negative advanced breast cancer, resulting in reduced toxicity and costs while maintaining efficacy [18]. A limitation of our study is that we assessed only the publication dates of evidence supporting SmPC changes or SmPC–guideline discrepancies, without considering the lag between publication and its incorporation into guidelines or the SmPC. A comparison of the current guideline and SmPC overlooks earlier SmPC changes incorporated into the guideline, as well as guideline updates later reflected in the SmPC. Furthermore, we did not evaluate the extent to which the post-authorisation studies sponsored by the MAH were mandated by the EMA. This was considered unfeasible because the Summary of Positive Opinion and the European Public Assessment Report only briefly mention additional studies requested post-authorisation. Detailed information on the required studies is specified in the Risk Management Plan (RMP), which has only been fully publicly available in full since 2023. Earlier versions of RMP summaries are not accessible, and publications by the MAH rarely specify that a study was conducted to fulfill post-authorization requirements.

The chosen timeframe allows sufficient opportunity for potential changes to occur. However, since attention to rational medicine use has only increased in recent years, these medicines, some of them already being off-patent by then, may have been studied less during that period [13]. In addition, not all SmPC changes or discrepancies could be linked to supporting evidence. For example, rituximab combined with chemotherapy for relapsed/refractory chronic lymphocytic leukemia (CLL) is recommended in the European guideline only for selected patients, and has largely been replaced by newer therapies. These restrictions in start criteria were likely driven by the emergence of new treatments rather than evidence showing benefit solely in this subgroup.

In conclusion, this study shows that the recommended use of biologicals changes significantly over the course of their phase of real world use. As most of these changes lead to decreased use of the biological, there is a gap between the market introduction of these medicines and their most rational use. Considerable effort and funding have gone into closing this gap. These studies were often initiated by public healthcare stakeholders, especially when they aim to investigate whether less use of a biological may be preferable. The substantial delay between marketing authorisation and the publication of these studies is concerning, as it may result in prolonged periods of suboptimal patient care and high societal costs.

The gap between the recommended use at introduction and the most rational use of the medicine will remain. When authorisation is granted based on limited evidence to allow early patient access, post-authorisation studies should be initiated by the MAH to further strengthen the evidence base. Efforts funded by public or charitable money should then be directed towards improving the rational use of medicines with a sufficiently established evidence base. These efforts should begin as soon as possible after marketing authorisation to ensure patients and society benefit from the rational use of these medicines.

## Supplementary Information

Below is the link to the electronic supplementary material.

Supplementary Material 1

Supplementary Material 2

Supplementary Material 3

## Data Availability

Collected study data will be made available to others when requested.
