# Supplementary material for: From authorisation to clinical practice: evolution of the use of biological medicines according to the SmPC and guidelines (2006 to 2025)
Source: Eur J Clin Pharmacol. 2026 Jun 10;82(7):165. doi: 10.1007/s00228-026-04097-5 (PMC13249682; doi:10.1007/s00228-026-04097-5)
Supplement: Supplementary file 1 — Supplementary Material 1 [file 228_2026_4097_MOESM1_ESM.docx]

**Supplementary text 1: Search methodology European clinical guidelines**

**Inclusion criteria:** Guidelines developed or endorsed by European professional societies or consortia. Most recent version available.

**Exclusion criteria:** Guidelines developed by non-European organizations or expert panels that do not reference a European professional society or consortium.

**Step 1:** Search via the website of the relevant European professional society.

**Step 2:** Systematic search on pubmed

**Pubmed search (**Example for Hunter syndrome)

("guideline"[Title] OR "guidelines"[Title]

OR "consensus"[Title] OR "consensus statement"[Title]

OR "recommendation"[Title] OR "recommendations"[Title]

OR "management"[Title]

OR "statement"[Title])

AND

("Hunter Syndrome"[Title/Abstract] OR "MPS2"[Title/Abstract] OR "Mucopolysaccharidosis type II"[Title/Abstract])
