# Supplementary material for: From authorisation to clinical practice: evolution of the use of biological medicines according to the SmPC and guidelines (2006 to 2025)
Source: Eur J Clin Pharmacol. 2026 Jun 10;82(7):165. doi: 10.1007/s00228-026-04097-5 (PMC13249682; doi:10.1007/s00228-026-04097-5)
Supplement: Supplementary file 2 — Supplementary Material 2 [file 228_2026_4097_MOESM2_ESM.docx]

**Supplementary table 1: Excluded biological-indication combinations**

| Biological | Disease | Specific indication at introduction | Reason for exclusion |
| --- | --- | --- | --- |
| Velaglucerase-alfa | Gaucher Disease | Type 1 Gaucher disease, long-term enzyme replacement therapy | No European guideline found |
| Romiplostim | Immune thrombocytopenic purpura (ITP) | Chronic immune thrombocytopenic purpura, splenectomised patients who are refractory to other treatments and patients where surgery is contra-indicated | No European guideline found |
| Epoetin alfa | Not applicable | For patients participating in an autologous blood donation program. Moderate anemia (Hb 6.2-8.1 mmol/L, no iron deficiency) if blood saving procedures are not available or insufficient when the scheduled elective surgery requires a large volume of blood (4 or more for women, 5 or more for men) | No European guideline found |
| Eculizumab | Paroxysmal nocturnal hemoglobinuria | Treatment of patients with PNH, evidence is limited to patients with history of transfusions | No European guideline found |
| Follitropin alfa and lutropin alfa | Luteinizing Hormone (LH) and Follicle Stimulating Hormone (FSH) deficiency | Stimulation of follicular development | No European guideline found |
| Human Normal Immunoglobulin | Immunodeficiency and immune-mediated diseases | Replacement therapy in PID syndromes, myeloma or chronic lymphocytic leukaemia with severe secondary hypogammaglobulinaemia and recurrent infections. Children with congenital AIDS and recurrent infections. ITP, Guillain-Barré, Kawasaki, Allogeneic bone marrow transplantation. | No European guideline found |
| Human normal immunoglobulin (IVIG) | Immunodeficiency and immune-mediated diseases | Replacement therapy in PID syndromes, myeloma or chronic lymphocytic leukaemia with severe secondary hypogammaglobulinaemia and recurrent infections. Children with congenital AIDS and recurrent infections. ITP, Guillain-Barré, Kawasaki, Allogeneic bone marrow transplantation | No European guideline found |
| Alemtuzumab | Chronic Lymphatic B-Cell Leukemia (B-CLL) | B-CLL patients for whom fludarabine combination chemotherapy is not appropriate | Marketing authorisation withdrawn |
| Bevacizumab | Breast cancer | Combination with doxetaxel chemotherapy as first line treatment for metastatic breast cancer | Marketing authorisation withdrawn |
| Rilonacept | Cryopyrin-Associated periodic syndromes (CAPS) | Cryopyrin-Associated periodic syndromes including FCAS and MWS | Marketing authorisation withdrawn |
| Ofatumumab | Chronic Lymphocytic Leukemia (CLL) | CLL, refractory to fludarabine and alemtuzumab. | Marketing authorisation withdrawn |
| Ibritumomab tiuxetan | Follicular Lymphoma | Consolidation therapy after remission induction in previously untreated patients with follicular lymphoma | Marketing authorisation withdrawn |
| Human hepatitis B immunoglobulin | Hepatitis B | Immunoprophylaxis against hep B | Marketing authorisation withdrawn |
| Peginterferon alfa-2b | Hepatitis C | Adult patients with compensated cirrhosis | Marketing authorisation withdrawn |
| Peginterferon alfa-2b | Hepatitis C | combination with ribavirin patients who have failed previous treatment with interferon alpha and ribavarin combination therapy or interferon alpha monotherapy. | Marketing authorisation withdrawn |
| Peginterferon alfa-2b | Hepatitis C | No previous treatment and clinically stable HIV-infection | Marketing authorisation withdrawn |
| Peginterferon alfa-2b | Hepatits C | Adult patients who have failed previous treatment with interferon alfa in combination with ribavirin. | Marketing authorisation withdrawn |
| Preotact recombinant human parathyroid hormone. | Osteoporosis | Postmenopausal women at high risk of fractures | Marketing authorisation withdrawn |

From authorisation to clinical practice: evolution of the use of biological medicines according to the SmPC and guidelines (2006 to 2025). European Journal of Clinical Pharmacology. B.M.F. Penninx1, C.E.M. Hollak, S.W. Tas, L. Timmers, S.J. de Visser, Z.L.E van Kempen

¹ Medicine for Society, Platform at Amsterdam University Medical Center, University of Amsterdam, Amsterdam, The Netherlands

[b.m.f.penninx@amsterdamumc.nl](mailto:b.m.f.penninx@amsterdamumc.nl)
