# Supplementary material for: From authorisation to clinical practice: evolution of the use of biological medicines according to the SmPC and guidelines (2006 to 2025)
Source: Eur J Clin Pharmacol. 2026 Jun 10;82(7):165. doi: 10.1007/s00228-026-04097-5 (PMC13249682; doi:10.1007/s00228-026-04097-5)
Supplement: Supplementary file 3 — Supplementary Material 3 [file 228_2026_4097_MOESM3_ESM.docx]

**Supplementary table 2: included biological-indication combinations**

| Biological | Disease | Specific indication at introduction | European Guideline |
| --- | --- | --- | --- |
| Abatacept | Rheumatoid arthritis | In combination with methotrexate for moderate to severe RA, in patients who had insufficient response or intolerance to other DMARDs including at least one anti-TNF agent. | [Smolen et al. Ann Rheum Dis., 2023](https://ard.bmj.com/content/82/1/3) |
| Adalimumab | Psoriatic arthritis | Active and progressive psoriatic arthritis in patients who have responded inadequately to DMARDS. | [Gossec et al. Ann. Rheum Dis, 2023](https://ard.bmj.com/content/83/6/706) |
| Adalimumab | Ankylosing spondylitis | Adults with severe ankylosing spondylitis who have had an inadequate response to conventional therapy | [Ramiro et al. Ann Rheum Dis., 2022](https://ard.bmj.com/content/82/1/19) |
| Adalimumab | Crohn's disease | Severe, active Crohn’s disease, in patients who have not responded despite a full and adequate course of therapy with a corticosteroid and/or an immunosuppressant; or who are intolerant to or have medical contraindications for such therapies. For induction treatment, Humira should be given in combination with corticosteroids. | [Gordon et al. J Crohns Colitis., 2020](https://academic.oup.com/ecco-jcc/article/18/10/1531/7693895?login=true) |
| Adalimumab | Psoriasis | Moderate to severe chronic plaque psoriasis in adult patients who failed to respond or have a contra-indication to other systemic therapies including cyclosporine, methotrexate or PUVA | [Nast et al. J Eur Acad Dermatol Venereol., 2020](https://onlinelibrary.wiley.com/doi/10.1111/jdv.16915) |
| Bevacizumab | Non small cell lung carcinoma | Unresectable advanced, metastatic or recurrent non-small cell lung cancer other than predominantly squamous cell histology, in combination with platinum-based chemotherapy | [Hendriks et al., Ann Oncol., 2023](https://www.annalsofoncology.org/article/S0923-7534(22)04785-8/fulltext) |
| Bevacizumab | Colorectal cancer | Metastatic colorectal disease, first line therapy, in combination with fluoropyrimidine based chemotherapy | [Cervantes et al., ESMO Metastatic Colorectal Cancer Living Guideline v 1.3](https://www.esmo.org/guidelines/living-guidelines/esmo-living-guideline-metastatic-colorectal-cancer) |
| Bevacizumab | Renal cell cancer | In combination with interferon alfa-2a, first-line treatment of patients with advanced or metastatic renal cell cancer | [Powles et al., Ann Oncol., 2024](https://www.annalsofoncology.org/article/S0923-7534(24)00676-8/fulltext) |
| Bevacizumab | Breast cancer | Metastatic breast cancer, first-line treatment in combination with paclitaxel or docetaxel | [Gennari et al., Ann Oncol., 2021](https://www.annalsofoncology.org/article/S0923-7534(21)04498-7/fulltext) |
| Canakinumab | Cryopyrin Associated Periodic Syndromes (CAPS) | CAPS including MWS, NOMID, CINCA, FCAS and FCU | [Romano et al., Ann Rheum Dis., 2021](https://www.sciencedirect.com/science/article/pii/S0003496724210177?via%3Dihub) |
| Certolizumab pegol | Rheumatoid arthritis | Moderate to severe active RA. In combination with MTx, when the response to DMARDs including MTx has been inadequate. Monotherapy in case of intolerance of methotrexate or when continued treatment with MTx is inappropriate. | [Smolen et al. Ann Rheum Dis., 2023](https://ard.bmj.com/content/82/1/3) |
| Cetuximab | Head and neck cancer | In combination with radiotherapy for locally advanced squamous cell cancer of the head and neck. | [Machiels et al., Ann Oncol., 2020](https://www.annalsofoncology.org/article/S0923-7534(20)39949-X/fulltext) |
| Cetuximab | Head and neck cancer | In combination with platinum-based chemotherapy for recurrent or metastatic disease for squamous cell cancer of the head and neck. | [Machiels et al., Ann Oncol., 2020](https://www.annalsofoncology.org/article/S0923-7534(20)39949-X/fulltext) |
| Cetuximab | Colorectal cancer | As a single agent in patients who have failed oxaliplatin and irinotecan based therapy and who are intolerant to irinotecant, EGFR expressing KRAS wild type metastatic colorectal cancer | [Cervantes et al., ESMO Metastatic Colorectal Cancer Living Guideline v 1.3](https://www.esmo.org/guidelines/living-guidelines/esmo-living-guideline-metastatic-colorectal-cancer) |
| Conestat-alfa | Angioedema | Acute angioedema attacks due to C1 esterase inhibitor deficiency. | [Maurer et al. Allergy., 2022](https://onlinelibrary.wiley.com/doi/10.1111/all.15214) |
| Corrifollitropine alfa | Subfertilty | Controlled ovarian stimulation in combination with a GnRh antagonist for the development of multiple follicles in women participating in an assisted reproductive technology program. | [Romualdi et al., Evidence-based guideline: unexplained infertility ESHRE., 2023](file:///C:\Users\P010832\Downloads\UI%20guideline_%20Final.pdf) |
| Denosumab | Osteoporosis | Bone loss associated with hormone ablation in men with prostate cancer at increased risk of fractures | [Coleman et al. Ann Oncol., 2020](https://www.sciencedirect.com/science/article/pii/S0923753420399956?via%3Dihub) |
| Denosumab | Osteoporosis | In postmenopausal women at increased risk of fractures | [Kanis et al., Osteoporosis Int., 2019](https://www.esceo.org/sites/esceo/files/pdf/Kanis%202019%20European%20guidance%20OI%2030%203.pdf) |
| Epoetin theta | Anemia | Symptomatic anaemia in patients with chronic renal failure | [Locatelli et al., Nephrol Dial Transplant., 2013](https://pubmed.ncbi.nlm.nih.gov/23585588/) |
| Epoitin theta | Anemia | Symptomatic anaemia in adult cancer patients with non-myeloid malignancies receiving chemotherapy | [Aapro et al., Ann Oncol., 2018](https://www.sciencedirect.com/science/article/pii/S0923753419316886?via%3Dihub) |
| Exenatide | Diabetes type 2 | Diabetes mellitus type 2, in addition to metformin and/or sulphonylurea, when no adequate glycaemic control is achieved on these medications | [Davies et al., Diabetes Care., 2022](https://link.springer.com/article/10.1007/s00125-022-05787-2) |
| Golimumab | Psoriatric arthritis | Alone or in combination with MTX, indicated for the treatment of active and progressive psoriatic arthritis in patients when the response to previous DMARD therapy has been inadequate. | [Gossec et al. Ann. Rheum Dis, 2023](https://ard.bmj.com/content/83/6/706) |
| Golimumab | Ankylosing spondylitis | Severe, active ankylosing spondylitis in adult patients who have responded inadequately to conventional therapy | [Ramiro et al. Ann Rheum Dis., 2022](https://ard.bmj.com/content/82/1/19) |
| Golimumab | Rheumatoid arthritis | In combination with methotrexate, indicated for moderate to severe active RA in patients when the response to DMARDS including MTX has been inadequate. | [Smolen et al. Ann Rheum Dis., 2023](https://ard.bmj.com/content/82/1/3) |
| Golimumab | Rheumatoid arthritis | Severe active rheumatoid arthritis, in combination with MTx, not previously treated with MTx | [Smolen et al. Ann Rheum Dis., 2023](https://ard.bmj.com/content/82/1/3) |
| Human hepatitis B immunoglobulin (Zutectra) | Hepatitis B | Prevention of HBV re-infection in HBV-DNA negative patients >6 months after liver transplantation | [EASL, J Hepatol., 2017](https://www.sciencedirect.com/science/article/pii/S016882781730185X?via%3Dihub) |
| Icatibant | Hereditary Angio Edema (HAE) | HAE, acute attacks in patients with C1 esterase deficiency. | [Maurer et al., Allergy., 2022](https://onlinelibrary.wiley.com/doi/10.1111/all.15214) |
| Idursulfase | Hunter Syndrome, MPS type 2 | Elaprase is indicated for the long term treatment of patients with Hunter syndrome (MPS type 2) | [Scarpa et al., Orphanet J Rare Dis. 2011](https://ojrd.biomedcentral.com/articles/10.1186/1750-1172-6-72) |
| Infliximab | Psoriatic arthritis | Active and progressive psoriatic arthritis in patients who have responded inadequately to DMARDS. In combination with MTx when possible | [Gossec et al. Ann. Rheum Dis, 2023](https://ard.bmj.com/content/83/6/706) |
| Infliximab | Ankylosing spondylitis | Treatment of severe active ankylosing spondylitis, in adult patients who have responded inadequately to conventional therapy | [Ramiro et al. Ann Rheum Dis., 2022](https://ard.bmj.com/content/82/1/19) |
| Infliximab | Crohn's disease | Crohn's disease, patients who have not responded despite a full and adequate course of therapy with a corticosteroid and an immunosuppressant. | [Gordon et al. J Crohns Colitis., 2020](https://academic.oup.com/ecco-jcc/article/18/10/1531/7693895?login=true) |
| Infliximab | Ulcerative colitis | Moderate to severe ulcerative coliltis, inadequate response to conventional therapy including corticosteroids and 6-MP or AZA, or who are intolerant to these medications. | [Raine et al. J Crohns Colitis., 2021](https://academic.oup.com/ecco-jcc/article/16/1/2/6390052?login=true) |
| Interferon beta-1b | Multiple sclerosis | Patients with RRMS and two or more relapses within the last two years. | [Montalban et al., Mult Scler. 2018](https://ectrims.eu/app/uploads/2024/01/ECTRIMS-EAN-Guideline-on-the-pharmacological-treatment-of-people-with-multiple-sclerosis-1.pdf) |
| Liraglutide | Type 2 diabetes mellitus | To achieve glycemic control in type 2 diabetes. In combination with metformin or a sulphonylurea or in combination with metformin or a sulphonylurea and a thiazolidinedione. | [Davies et al., Diabetes Care., 2022](https://link.springer.com/article/10.1007/s00125-022-05787-2) |
| Methoxy polyethylene glycol-epoetin beta | Anaemia associated with chronic kidney disease | Anemia associated with CKD | [Locatelli et al., Nephrol Dial Transplant., 2013](https://pubmed.ncbi.nlm.nih.gov/23585588/) |
| Natalizumab | Multiple sclerosis | Single DMD therapy in highly active relapsing remitting multiple sclerosis, despite treatment with beta-interferon or patients with rapidly evolving severe relapsing remitting MS. | [Montalban et al., Mult Scler. 2018](https://ectrims.eu/app/uploads/2024/01/ECTRIMS-EAN-Guideline-on-the-pharmacological-treatment-of-people-with-multiple-sclerosis-1.pdf) |
| Panitumumab | Colorectal cancer | EGFR expressing colorectal cancer with non-mutated KRAS after failure of fluoropyrimidine, oxaliplatin and irinotecan containing chemotherapy regimens. | [Cervantes et al., ESMO Metastatic Colorectal Cancer Living Guideline v 1.3](https://www.esmo.org/guidelines/living-guidelines/esmo-living-guideline-metastatic-colorectal-cancer) |
| Ranibizumab | Neovascular wet age-related macular degeneration | Age-related wet macular degeneration (initiation). Loss greater than 5 letters in visual acuity (maintenance phase) | [Schmidt-Erfurth et al., Br J Ophthalmol., 2014](https://bjo.bmj.com/content/98/9/1144.long) |
| Ranibizumab | Diabetic Macular Oedema | Visual impairment due to diabetic macular oedema | [Schmidt-Erfurth et al., Ophthalmologica., 2017](https://pubmed.ncbi.nlm.nih.gov/28423385/) |
| Recombinant human acid alpha-glucosidase | Pompe's disease | Long-term ERT in patients with confirmed diagnosis of Pompe disease. Benefits of myozyme in patients with late-onset Pompe disease have not been established. | [Schoser et al., Eur J Neurol., 2024](https://onlinelibrary.wiley.com/doi/10.1111/ene.16383) |
| Rituximab | Rheumatoid arthritis | In combination with MTx for the treatment of severe active RA, inadequate response or intolerance to other DMARDs including one or more anti-TNF agents | [Smolen et al. Ann Rheum Dis., 2023](https://ard.bmj.com/content/82/1/3) |
| Rituximab | Follicular lymphoma | Maintenance therapy for relapsed/refractory follicular lymphoma that responds to induction therapy with chemotherapy with or without rituximab | [Dreyling et al. Ann Oncol., 2021](https://www.annalsofoncology.org/article/S0923-7534(20)43163-1/fulltext) |
| Rituximab | Follicular non-hodgkin lymphoma | Stage III-IV follicular lymphoma, untreated patients, in combination with chemotherapy | [Dreyling et al. Ann Oncol., 2021](https://www.annalsofoncology.org/article/S0923-7534(20)43163-1/fulltext) |
| Rituximab | CLL | In combination with chemotherapy for patients with relapsed/refractory CLL | [Eichhorst et al., Ann Oncol., 2021](https://www.annalsofoncology.org/article/S0923-7534(20)42469-X/fulltext) |
| Rituximab | CLL | First line therapy of CLL in combination with chemotherapy | [Eichhorst et al. Ann Oncol., 2024](https://www.annalsofoncology.org/article/S0923-7534(24)00747-6/fulltext) |
| Rituximab | Follicular Lymphoma | Maintenance therapy for previously untreated follicular lymphoma that responds to induction therapy. | [Dreyling et al., Ann Oncol., 2021](https://www.annalsofoncology.org/article/S0923-7534(20)43163-1/fulltext) |
| Teriparatide | Osteoporosis in men | Treatment of osteoporosis in men at increased risk of fracture. | [Fuggle et al., Nat Rev Rheumatol., 2024](https://www.nature.com/articles/s41584-024-01094-9#Sec22) |
| Thyrotropin alfa | Thyroid cancer | Pre-therapeutic stimulation in low-risk post-thyroidectomy patients maintained on hormone suppression therapy for the ablation of thyroid remnant tissue in combination with radioactive iodine | [Filetti et al., Ann Oncol., 2019](https://www.sciencedirect.com/science/article/pii/S0923753420325552?via%3Dihub) |
| Tocilizumab | Rheumatoid arthritis | In combination with MTX for moderate to severe RA, patients who have responded inadequately to or were intolerant to previous therapy with one or more DMARDs or TNF antagnoists. | [Smolen et al. Ann Rheum Dis., 2023](https://ard.bmj.com/content/82/1/3) |
| Trastuzumab | Gastric or GEJ cancer | Metastatic gastric or GEJ HER+ adenocarcinoma, in combination with capecitabine or 5-FU and cisplatin. | [Lordick et al., Ann Oncol., 2022](https://www.annalsofoncology.org/article/S0923-7534(22)01851-8/fulltext) |
| Trastuzumab | Breast cancer | Adjuvant treatment of HER2+ early breast cancer following (neo-)adjuvant chemotherapy and if applicable radiotherapy, in combination with taxanes | [Loibl et al., Ann Oncol., 2023](https://www.annalsofoncology.org/action/showPdf?pii=S0923-7534%2823%2905104-9) |
| Trastuzumab | Breast cancer | IN combination with docetaxel for the treatment of those patients who have not received chemotherapy for their metastatic disease | [Gennari et al., Ann Oncol., 2021](https://www.annalsofoncology.org/article/S0923-7534(21)04498-7/fulltext) |
| Ustekinumab | Psoriasis | Moderate to severe plaque psoriasis, failed to respond to or have a contra-indication or are intolerant to other systemic therapies including ciclosporin, methotrexate and PUVA | [Nast et al. J Eur Acad Dermatol Venereol., 2020](https://onlinelibrary.wiley.com/doi/10.1111/jdv.16915) |

From authorisation to clinical practice: evolution of the use of biological medicines according to the SmPC and guidelines (2006 to 2025). European Journal of Clinical Pharmacology. B.M.F. Penninx1, C.E.M. Hollak, S.W. Tas, L. Timmers, S.J. de Visser, Z.L.E van Kempen

¹ Medicine for Society, Platform at Amsterdam University Medical Center, University of Amsterdam, Amsterdam, The Netherlands

[b.m.f.penninx@amsterdamumc.nl](mailto:b.m.f.penninx@amsterdamumc.nl)
